# Comparison of clonidine and dexmedetomidin influence on the main indicators of hemodynamic during induction of anesthesia in neurooncological patients

**DOI:** 10.1186/2197-425X-3-S1-A988

**Published:** 2015-10-01

**Authors:** R Nazarov, A Kondratiev, M Rumiantseva, A Petrova

**Affiliations:** Federal State Institution 'North-West Federal Research Center' of the Russian Ministry of Health, Anesthesiology and Intensive Care St. Petersburg, Russian Federation; Federal State Institution 'North-West Federal Research Center' of the Russian Ministry of Health, St. Petersburg, Russian Federation

## Introduction

opioid and adrenergic antinociceptive systems are the components of neuroregulatory system of a brain stem. The impact on neuroregulatory systems is a point of theoretical and practical interest for neuroanesthesia. We successfully apply the method of anesthesia, which includes combined impact on opioid (fentanyl) and adrenergic (clonidine) antinociceptive systems. This method of anesthesia creates conditions for neurovegetative stability which are optimal for brain surgery.

Emergence of a more selective alfa2-adrenoagonist (Dexmedetomidin), allows to consider it as choice option for neurovegetative stabilization.

## Objectives

To compare effects of Clonidine and Dexmedetomidin on the main indicators of hemodynamic during induction of anesthesia in neurosurgery.

## Methods

In the research we included 63 patients who underwent operations on posterior brain fossa and pineal area. At all patients the aneshtesiological maintenance included: miorelaxant (pipekuronium 0,1 mg/kg or rokuronium 0,6 mg/kg), hypnotic (propofol 2 mg/kg), opioid analgetic (fentanyl of 3,54-5,88 mkg/kg) + alfa2-adrenoagonist (clonidine and dexmedetomidin). All the patients were divided into two groups: in the 1-st group (21 patient) patients received Clonidine 1-2,63 mkg/kg, in the 2-nd group (42) - Dexmedetomidin 0,88-2,33 mkg/kg.

Monitoring of the main hemodynamic indicators (systolic/ diastolic blood pressure, heart rate) was carried out by the device "Nihon Kohden". All operations were performed under control of the sedation depth by the device "BIS Aspect".

## Results

In the 1-st group all the patients (21) demonstrated the reduction of the heart rate after introduction of the anesthesia by 7,7 - 50% from a reference value. In 1 case (4,8%) the increase of systolic AP (arterial pressure) for 11,5% and the diastolic AP for 31,2% of reference value was noted. In other 20 cases (95,2%) there was a decrease of systolic AP for 7,1 - 48,1% and the diastolic AP for 12,5 - 55% . In the2-nd group all 42 patients demonstrated the decrease of the heart rate by 18,2 - 61,5% after the introduction of anesthesia. In 34 cases (80,9%) the increase of systolic AP for 0 - 58,3% (average value 21 ± 12,7%) and the diastolic AP for 0 - 57,1% from reference values was noted. In other 8 cases (19,1%) there was a decrease of systolic AP for 16,1 - 36,7% and diastolic AP for 10 - 38,1%.

In all 63 cases these changes were observed in the conditions of the sufficient depth of sedation by BIS (15-30).

## Conclusions

Thus, when using Clonidine for neurovegetative stabilization, in 95,2% of cases the central simpatolytic effect causing decrease in heart rate and AP prevailed. And in cases with Dexmedetomidin's application in 80,9% of patients after an introduction anesthesia the peripheral vasoconstrictive effect, causing the increase of system vascular resistance and AP with further decrease in heart rate, prevailed.

